# Moral landscapes and morally meaningful encounters: how interaction ritual connects conversation analysis and cultural sociology

**DOI:** 10.3389/fsoc.2024.1251164

**Published:** 2024-04-25

**Authors:** Mervyn Horgan

**Affiliations:** Department of Sociology & Criminology, University of Guelph, Guelph, ON, Canada

**Keywords:** interaction ritual, civil inattention, cultural sociology, moral landscapes, Durkheim, Goffman, public space

## Abstract

This article presents a theoretical argument for examining the previously unexamined interface between the strong program in cultural sociology ethnomethodology/conversation analysis (EMCA). While these two approaches have radically different theoretical and empirical commitments, they nonetheless share a common root in Durkheim’s sociology, specifically with regard to the centrality of solidarity, ritual, and morality to collective life. Similarly rooted in Durkheim, Goffman’s theory of interaction ritual provides an analytic pivot between EMCA and the strong program. The broader theoretical argument is illustrated using data from interviews with adults about their most recent encounter with a rude strangers in public space, which are here treated a breaches of the interaction ritual of civil inattention. Members readily draw on the specifics of a particular stranger interaction gone awry to reflect on the nature of life in public and to expound on their understandings of the ethics of face-to-face interaction and everyday morality more generally. Where EMCA focuses on the discoverability of the organizational features of everyday interaction, the position developed here is concerned with the organization of members’ interpretations of everyday interaction. While centered on specific kinds of interactional breaches, by finding common ground between EMCA and cultural sociology, the argument advances a potentially more broadly applicable approach that treats everyday encounters as morally meaningful and everyday lifeworlds as moral landscapes. Developing a comprehensive understanding of copresent interaction as a basic building block of society requires attention to both the organizational dynamics of copresent encounters and to the interpretive resources that ordinary members use to account for and justify their own and others’ conduct.

## Introduction

‘I’m always friendly to strangers. Everyone should be. Until they give a reason not to be.’

-Julia, white female, early 20s.

Encounters between strangers are rarely fatal, but often fateful. As spaces populated predominantly by people unknown to one another, the dominant interactional form in public space is between strangers. That order is produced and maintained in public spaces makes them sociologically generative. Where only the most basic common ground is shared, interactions between strangers generally proceed unproblematically. The urban interaction order is a moral order, and this is highlighted when stranger interactions go awry.

Building on the ethnomethodological-conversation analytic tradition of treating breaches as analytically generative, this article treats rude encounters between strangers in public space as breaching a specific interaction ritual: civil inattention (Goffman, 1963). While inspired and informed by the spirit of EMCA, to be clear from the outset, this article does not ‘do’ EMCA. Where EMCA focuses on the discoverability of the *organizational features* of everyday interaction (Maynard and Clayman, 1991), here my argument centers on the *organization of members’ interpretations* of everyday interaction. Where EMCA approaches focus on the “structural organization of social interaction” (Stivers, 2015, p. 1), to make a theoretical argument, I draw on a major movement in contemporary sociological theory—the ‘strong program in cultural sociology’ (Alexander and Smith, 2001)—to show how members’ own reported *post hoc* interpretations of encounters gone awry give us new theoretical purchase on the moral meanings that members attach to everyday interactions.

While the argument is primarily theoretical, to provide some empirical footing I draw on a database of interviews about encounters with ‘rude’ or ‘uncivil’ strangers in public space, looking in particular at how participants justify incivility. In discussing incivilities, interview participants treat encounters with strangers in public space as morally meaningful. These meanings are malleable, but nonetheless, structured. Drawing on the specifics of stranger encounters gone awry, participants reflect on collective life in public space, expounding their understandings of the ethics of face-to-face interaction and everyday morality. Treating everyday lifeworlds as moral landscapes advances a perspective that probes common ground between EMCA and cultural sociology, though with potentially broader applicability than either approach. All encounters may be morally meaningful, but some bear greater moral density, and for members, are readily tethered to deep structures of meaning. What, then, do we learn not just from stranger encounters gone awry, but from members’ accounts of such blips and bumps in the urban interaction order? It is my contention here that to develop comprehensive understanding of copresent interaction as a basic building block of society, we must attend both to the organizational dynamics of copresent encounters *and* to the interpretive resources that ordinary members use to account for and justify their own and others’ conduct. This is a relatively delicate theoretical point and requires attention to both EMCA and cultural sociology.

## Never the twain? EMCA and cultural sociology

“A multitude of myopias limit the glimpse we get of our subject matter” (Goffman, 1983, p. 2).

To set the scene for the theoretical argument we can ask: what happens when we bring similarly rooted but widely divergent perspectives together? Informed by both EMCA and the strong program, I nudge both approaches out of their respective wheelhouses to take a new tack on previously analyzed phenomena. Where building barricades and defending turf limits disciplinary innovation, cross-fertilization can advance our common enterprise. Probing points of overlap between EMCA and cultural sociology means identifying not only shared elements of each approach, but also areas of productive tension: ‘normal science’ (Kuhn, 1962) this is not. For Garfinkel, “a consistent application of ethnomethodology rejects all forms of sociological generalization” (Tavory, 2022, p. 42), but if we wish to find ways to reconnect EMCA and sociological theory, possibilities for generalization should remain on the table.

EMCA is conceptually grounded in Durkheim’s sociological theory (Garfinkel, 2002; Rawls, 2012, 2022). It is informed by a Durkheimian conception of social order, centered in part on ritual, morality, and the sacred status of both persons and the collective as anchors for social solidarity. While Durkheim identified and analyzed the moral order, “the exteriority and constraint of a given moral order did not await his writing for its appreciation or characterization by those subject to its influence” (Wilson and Zimmerman, 1979, p. 55). Informed by Durkheim’s late work (in particular, *The Elementary Forms of Religious Life*), cultural sociology has blossomed over the last quarter century. Despite many surface differences, resonance—and dissonance—between EMCA and the strong program in cultural sociology are legion. Significantly, both approaches are centrally concerned with solidarity. EMCA treats the collaborative production of locally situated social order as an intersubjectively upheld moral order (Schegloff, 1992; Rawls, 2010). The ‘strong program’ treats ritual, morality, and the symbolic codes of sacred and profane as constitutive features of social solidarity. Examining resonances between these approaches is part of a broader project probing ways that analyses of interaction and of symbolic dimensions of collective life might learn from, enhance, and mutually reinforce one another [see Horgan (2019, 2020, 2021, Forthcoming)].

In the quote that opens this section Goffman does not claim to have the corrective analytic lens for our ‘myopias.’ He suggests that focusing exclusively on the interaction order is one way to develop sociological insight. Goffman centers analytic attention on the endogenous organization of social order among copresent persons. EMCA practitioners have productively plowed this terrain for over a half century, all the while distinguishing their approach from Goffman’s. While “relations between EMCA and Goffman have been complicated during Goffman’s life and in the later reception of his work” (Mondada and Peräkylä, 2024, p. 2) and “relations between Goffman and ethnomethodology are complex” (Inglis and Thorpe, 2023, p. 6), the theoretical argument here uses Goffman’s work to open up dialog between EMCA and sociological theory. Centering Durkheimian elements in Goffman’s (1963, 1967, 1971) sociology—interaction ritual in particular—provides an analytic pivot point.

Sociologies of ritual and morality are inextricably linked. “Goffman, Garfinkel, and Schegloff treat the grounding of interaction as essential to the sociology of morality” (Tavory and Fine, 2020, p. 369) and through the work of Durkheim and Goffman “the sociology of morals, was realized in the form of a sociology of rituals” (Bergmann, 1998, p. 282). For Smith G. (2022, p. 49), Goffman’s interaction ritual opens up “the sociologically unexamined moral weight of our words, glances and gestures,” making it a useful bridge between EMCA and cultural sociology. For Wuthnow (1989, p. 101), Goffmanian ritual is not “a type of social activity that can be set off from the rest of the world for special investigation. It is a dimension of all social activity. The study of ritual, therefore, is not distinguished by its concern with certain types of activity, but by the perspective it brings to bear on all activity, namely, emphasis on the symbolic or expressive dimension of behavior.” This approach understands interaction’s ritual dimensions as holding specifiable expressive potential and qualities. This differs from a more strictly EMCA approach that sidesteps “*ritual* constraints on interaction…. and focuses more on *system* constraints” [Maynard, 2012, p. 17, emphases in original; see also Mondada and Peräkylä (2024)]. Nonetheless, Alasuutari (2023, p. 1) suggests “what CA calls institutional interaction should be considered as rituals.” Although EMCA has tended to avoid ritual language or framing, rituals both constitute and express norms, and reinvigorate and manipulate shared symbols. Centering everyday ritual then is one way—though the only one—to get at everyday morality. Ritual provides a dramatic structure for interaction (Burke, 1969; Turner, 1995; Tavory and Fine, 2020). Centering ritual dimensions of interaction permits attention both to the internal organization of the interaction order and the strong program in cultural sociology’s more macro-oriented proclivities.

While discoveries by EMCA practitioners exploring the endogenous organization of the interaction order over the last half century have been remarkable, less progress has been made in understanding the “loose coupling” (Goffman, 1983, p. 11) between what happens within the interaction order and broader structures of meaning that persons draw upon, enliven, and deploy in their experiences and interpretations of everyday interaction. EMCA helps in understanding the exclusively interactional end of this loose coupling. To connect to broader structures of meaning we need a wider lens. Where EMCA examines in ever more empirical detail the inner space of the interaction order, my argument is more theoretical in nature, suggesting that the endogenous organization of the interaction order while important, needs also to be understood in terms of how members’ interpretations of that organization are tethered to and hang upon more generally available and intelligible structures of interpretation, what we might call, moral narratives. Such narratives posit ideals of conduct with high symbolic charge.

My tack on the loose coupling, then, is to connect the interaction order to the cultural structures that both infuse this order and animate its’ broader conditioning environments. Sociologically, my reasoning for this is; (1) everyday interaction is largely overlooked in strong program cultural sociology, and (2) EMCA has yet to develop a satisfactory theory of culture.^1^ The next section elaborates on these absences by first outlining weaknesses in the strong program in cultural sociology (Alexander and Smith, 2001) vis-à-vis the interaction order, before turning to select EMCA work explicitly addressed to culture. Following this, I highlight research suggesting possibilities for mutual reinforcement between cultural sociology and EMCA.

### Cultural sociology absent the interaction order

The strong program in cultural sociology has emerged over the last quarter-century as a conceptually-driven, theoretically generative, hermeneutically-nuanced approach. Specifically, by giving “relative autonomy” to culture, the strong program investigates how culture shapes “actions and institutions, providing inputs every bit as vital as more material or instrumental force” (Alexander and Smith, 2001). Drawing directly on Durkheim, culture is granted *causal power* in social life, specifically Durkheim’s clarification of what he takes to be the core subject matter of sociology—*conscience collective* and collective representations—the objects toward which a science of society should be addressed: “collective representations convey…the way in which the group conceives itself in its relations to objects which affect it” (Durkheim, 1964, p. 49). Drawing this insight together with Durkheim’s (1995) later elaborations, (Alexander and Smith, 2005; Smith, 2020), the strong program examines how *conscience collective*, the binding force of collective morality and solidarity, operates through binary structured discourse. Refining this further, Alexander (2006) shows how actors mobilize the “binary discourse of civil society” to evaluate motives, actions, and institutions. While specific mobilizations of these discourses vary contextually, because of its relative autonomy, the structure of the discourse itself—its’ symbolic power—is stable. The task for cultural sociologists, then, is to show what these symbolically-laden discourses do, how they are invoked and mobilized in expanding, contracting, or shifting solidarities. In short, how they interpretively consolidate or re-align affiliation and disaffiliation.

Anchored in a core strategy of granting analytic autonomy to culture, on this view, culture is instantiated within the interaction order, but retains a relatively stable structure independent of its’ iteration in any given interaction order. Consequently, the approach remains relatively undergrounded in terms of taking seriously everyday lifeworlds and the quotidian interaction order. Instead, cultural sociologists tend to concentrate on crises, societal tensions, scandals, largescale social movements, or rapid and radical social transformation. Rather than taking the interaction order as an internally structured, endogenously organized reality, it is treated as a mere settings where symbolic codes—sacred/profane, pure/impure, for example—manifest. Thus, cultural sociology remains largely silent on how codes are invoked, reinforced, and constituted in and through the mundane interaction order as a lived embodied copresent reality. While EMCA scholars do not need to be reminded of the significance of mundane interaction, sometimes cultural sociologists do. Where cultural sociology treats *culture* as analytically autonomous, EMCA treats the *interaction order* as analytically autonomous.

### Cultural internalism: EMCA’s interactionally grounded and bounded theory of culture

Within EMCA, the constant refinement of analysis and ongoing discovery of ever more themes and variations within the basic universal structure of turn-taking, sequencing, and repair are and will continue to generate significant insights. That said, read alongside advances in cultural sociology, EMCA conceptualizations of culture are relatively underdeveloped. For Schegloff (2006, p. 70), social interaction is “the arena in which culture is enacted,” but this enactment is restricted. If the interaction order is wholly autonomous, then culture is only empirically available within the operations of the bounded interaction order. While deriving analytic power from unwavering commitment to analyses of naturally occurring interaction, the view of culture while interactionally grounded, is also interactionally bounded.

Nonetheless, intimations of connections to cultural sociology exist within EMCA scholarship. To understand the diagnostic encounter between doctor and patient, Bergmann draws on and extends analysis beyond a strictly EMCA frame, toward the broader ideological context that grants power to psychiatry. This provides a cultural diagnosis of psychiatry itself.^2^ Building on Sacks’ membership categorization analysis, Hester argues that there is little support for a culturalist view of category use, particularly in institutional talk [see Francis and Hester (2017)]. The main objection here is the decontextualization of interactional practices by imposing a “‘stable’ cultural framework” (Francis and Hester, 2017, p. 58) on data. This aligns with a deeper issue with how ‘culture’ is conceptualized in EMCA primarily in the traditional anthropological sense of language and ways of life shared by bounded wholes. For example, studying American and Thai conversational practices, Moerman (1988, p. 4) uses cross-cultural materials to propose that “sequential organization be used to locate, describe, and provide a metric for cultural variation”.^3^ Generally then, EMCA’s theory of culture tends to either (i) note ‘cultural variation’ across different linguistic contexts and institutional settings or (ii) seek universals across cultures (Levinson, 2006; Stivers et al., 2009; Dingemanse et al., 2015).

Granting analytic autonomy to the interaction order is endlessly generative: it carves off methodologically digestible chunks of intersubjectively produced social reality for scrutiny. Problems with EMCA conceptions of culture derive from slippage between EMCA’s epistemology—the *analytic* autonomy of the interaction order—and its’ broader social ontology—treating the interaction order as wholly *empirically* autonomous. On this view, the interaction order is not just a slice of social reality to be carved off for analysis, elevated instead to the sole constitutive feature of social life, and thus the sole object worthy of sociological scrutiny (Rawls, 2009). Taking this analytic strategy as the totality of social reality both limits the range of available conceptual resources and methodologies, and prevents potentially relevant phenomena from surfacing. Thus, EMCA’s cultural internalism is both a core analytic strength and, read through a cultural sociological lens, a significant lacuna. While highly refined internally, EMCA is also characterized by rigid boundary maintenance limiting its engagement with social theory more generally. The theoretical argument here takes an openly skeptical stance toward what Kendon (1990) calls the “natural history” tradition of interaction studies.

Earlier, Sacks (1995, p. 226) intimated possibilities for a somewhat more expansive conceptualization of culture: “a culture is an apparatus for generating recognizable actions.” From this, one area of focus in EMCA is on the production of such recognizability within the interaction order. While ‘apparatus’ may connote a mechanistic model, we can posit that if culture is the apparatus, then *interpretation* is the activity that generates recognizability. The production of recognizability necessitates that members draw upon readily available and intelligible structures of meaning and interpretation. An interpretation that is intelligible *within* a particular interaction order must also be at least partially intelligible *outside* that specific context of interaction. My approach suggests one way to move between this inside and outside. EMCA helps us with the inside, and, in the spirit of this special issue’s theme, for the outside, reconnecting EMCA to sociological theory is germane.

Overall, both EMCA and cultural sociology, use homologous analytic strategies: treating their objects—for EMCA, the interaction order; for cultural sociology, symbolically coded cultural structures—as analytically independent entities. For both, ‘independence’ means autonomy from other spheres of collective life, for example politics, or the economy. While these other spheres are, of course, also where the interaction order figures as a constitutive feature and is imbued with symbolic codes, both cultural sociologists and EMCA practitioners necessarily focus analytic attention on specific slices carved off from the whole of social reality. Undoubtedly, deep analytic tensions exist between a perspective centered exclusively on copresent persons’ interactional practices, and one that views social life as organized around relatively stable symbolic codes unbounded by any specific scene of copresent interaction. Treating these tensions as productive, by examining common occurrences in everyday life—mundane breaches, those “petty annoyances” (Smith et al., 2010) of rudeness or incivility between strangers, to which we will soon turn—we can look to how EMCA and cultural sociology might inform one another in analyzing collective life’s specifically moral dimensions.

Thinking at the intersections of cultural sociology and EMCA means brushing against a range of adjacent literatures. Next, I briefly survey literatures intimating connections between cultural sociology and EMCA, before turning to a discussion of the place of ritual in the argument and illustration that follows.

### Building bridges

While cultural sociologists tend to focus on collective representations, media discourses, and political performances, there are some strands of cultural sociological scholarship that engage with mundane interaction. Similarly, despite the critiques of EMCA outlined above, there are some promising tendencies in broadly adjacent work. Below I quickly review work that, to various degrees, resonates with both EMCA and cultural sociology. This work can be characterized as culturally-attuned qualitative research connecting interactional practices and structures of meaning.

There are a variety of tendencies here, with much research focusing on interaction in institutions—for example, schools, workplaces, and the domestic sphere (Willis, 1981; Blair-Loy, 2009; Lareau, 2011)—with a view to understanding the role played by cultural ideals in social reproduction in general, and inequality in particular (Schwalbe et al., 2000; Valentino and Vaisey, 2022). Others examine how cultural representations intervene at the scene of interaction, for example, how pervasive images of the ‘iconic ghetto’ shape interracial encounter in the US (Anderson, 2023), or how public health concerns around HIV/AIDS and condom use appear in intimate encounters (Tavory and Swidler, 2009). Rapprochement between interaction-focused approaches and cultural sociology also advance understanding of the multiple drivers of political polarization at the level of personal relations (Revers, 2023). In other ethnographically-grounded approaches, work on “culture in interaction” looks at “how groups put culture to use in everyday life” (Eliasoph and Lichterman, 2003, p. 735), with a view to understanding possibilities for “civic action” (Lichterman and Eliasoph, 2014; Lichterman, 2020).

Also relevant are studies of patterns of interaction in group life, some EMCA-adjacent and some closer to culturally-attuned sociological social psychology. Broadly conceived, these approaches reach toward more general social theory. Informed by EMCA, DeLand (2021) incorporates analysis of character and biography into ethnographies of group activity. Work in interactional pragmatics and the broader multimodal turn advances understandings of the local accomplishment of intercultural communication and competency in public settings and in periods of crisis (Mondada, 2009; Mondada et al., 2020). Similarly, recent work unpacks relationships between interaction troubles and the broader structures of meaning that members draw upon in their accounts (Stevanovic et al., 2023). Further, studies of idioculture, group life, and local action are closely aligned with elements of cultural sociology, while attending closely to copresent interaction (Fine and Fields, 2008; Fine, 2010, 2012; Corte et al., 2019; Rawls and Turowetz, 2021). Save for two exceptions (Anderson, Mondada), one key difference between these studies and the present argument is that most attempts to connect the interaction order and structures of meaning center on pre-existing groups, where persons are already connected in some way, for example, in schools, workplaces or households. EMCA has not dealt extensively with stranger interactions.^4^ For example, while Bergmann’s (1993) work on gossip does attend to culture, gossip is an interactional practice that depends on existing relations and sedimented interactions between people known to one another. When it comes to interactions between strangers in public space, no pre-existing groupedness can be assumed.

## Interaction: ritual and breach

Goffman’s (1963, 1971) sustained analysis of interaction in public spaces connects clearly to a central concern of social theory: how is social order accomplished where persons are unknown to one another? While the production and reproduction of social order occurs *in* interaction, it depends too on generalizable principles operating across situations and contexts. For both Durkheim and Goffman, *ritual* is the vital social modality through which the sacred is given form: ritual constitutes, expresses, and renews the sacred. While conceptions of *what* is sacred vary, *how* that sacred is produced is consistent. Ritual provides a shared focus that creates and renews group members’ binds to a collective (Durkheim, 1995). This finds its apex in the fleeting production of collective effervescence: ritual and solidarity are inseparable.

While Goffman’s (1963, 1967, 1971) interaction ritual hews close to Durkheim, it goes beyond formal ritual: Goffman’s innovation brings Durkheimian ritual to everyday life.^5^ For Durkheim, a core mechanism in the production of solidarity involves “micro-level ritualized encounters in which members plunge themselves in the ‘waters’ of the group and renew their commitment…. These mechanisms serve two purposes: to ensure the reproduction of social life, supplying individuals with meaning and purpose and collectives with motivated actors and second, as protective forces against acute blows to the collective, whether endogenous or exogenous” (Abrutyn, 2022). Where, for Durkheim, ritual marked special occasions and moments of heightened group solidarity, Goffman treats ritual as a core feature of everyday interaction. Interaction between copresent persons display ritual elements through which the sacred status of persons and of the collective can be confirmed or disconfirmed. Thus viewed, ritual is intrinsic to everyday life. Wuthnow (1989, p. 109) defines ritual as “*a symbolic-expressive aspect of behavior that communicates something about social relations, often in a relatively dramatic or formal manner*.” (emphasis in original). In discussing Goffman’s work on ritual, Wuthnow proposes that we take ritual as a *dimension* of all social activity, where “[t]he regulation of daily life...depends on ritual and, for this reason, is imbued constantly with the ritual dramatization of symbolic meanings” (102). Ritual structures interpersonal encounter: members are charged with conducting themselves in contextually appropriate ways. Interaction ritual, though, cannot be reduced to mere rules of conduct, instead it provides a “guide for actions, recommended not because it is pleasant, cheap, or effective, but because it is suitable or just” (Goffman, 1956, p. 473). Thus, for Goffman, ritual elements organize the accomplishment of social order, with the internal organization of any given interaction order partly dependent on members sharing a general understanding and ritual commitment. Examples are myriad throughout his oeuvre; in ‘Deference and Demeanor’ Goffman (1967, p. 47) shows the interactional work required to attend to externally granted but internally active status characteristics and status differences among interactants. Notably, this essay opens by discussing Durkheim’s sociology, and observes that “the rites performed to representations of the social collectivity will sometimes be performed to the individual himself [*sic*].”

Ritual, then, is instrumental in the sense that it permits the everyday business of interaction to proceed in relatively conventionalized and mutually intelligible ways (Terkourafi and Kádár, 2017). More importantly for the present argument, ritual *communicates*: it is expressive. Precisely *what* ritual expresses is—in the spirit of Durkheim’s social pathology, Garfinkel’s breaches, and Goffman’s situational improprieties—best accessed through its rupture. Inspired by this foundational approach in EMCA, I treat breaches as instructive not only regarding the local accomplishment of social order, but also as objects that members readily connect to broader questions of morality and solidarity.

### Strangers and public space: civil inattention as ritual, incivility as breach

With global mobility and intense urbanization bringing more and more strangers into ever closer proximity, interactions between strangers are the most ubiquitous form of interaction on earth [see Arminen and Heino (2023)]. While large in number, strangers’ public interactions differ qualitatively from interactions in other settings, from interactions between persons known to one another, and between persons in defined roles. In the absence of more specific common ground, stranger encounters lean heavily on ritualized interaction (Ickes, 2009). Goffman (1963) develops the concept of civil inattention as “the slightest of interpersonal rituals” essential to the accomplishment of order in public interactions among strangers. Drawing on Durkheim’s (1995) distinction between positive and negative rites, Goffman (1967, p. 73) differentiates presentational rituals and avoidance rituals in interaction. As negative rites, avoidance rituals are about what a person must *not* do in order to respect the rights of another. In the Goffmanian idiom then, civil inattention is an avoidance ritual.

Our interest is in breaches of this specific ritual. First though, it is important to note that my treatment of breaches aligns less with Garfinkel’s (1967) storied experiments than it does with Durkheim and Goffman. For Garfinkel, by querying common sense understanding and expectancies around the reciprocity of perspectives, breaches highlight how sense-making and intelligibility unfold in locally situated interaction. In contrast, I treat breaches as ruptures in interactional norms that draw attention to the more broadscale production of social order, and that members do not orient to as exclusively locally situated products of interaction.^6^

Civil inattention raises analytic questions for both EMCA and cultural sociology. It is one among many of the “norms of co-mingling” (Goffman, 1971, p. 9), but as an interaction ritual, it is also something more. The common ground shared by copresent strangers is of the most general kind: being in the same place at the same time (Goffman, 1963; Simmel, 1971; Lofland, 1973, 1998; Smith R. J., 2022). In analyzing densely populated settings shared by copresent persons where civil inattention prevails, we cannot assume the groupedness of such aggregations of persons. Conduct in public spaces is different from other contexts. Distinct, for example, from the private realm of intimacy or workplaces, where shared orientation, existing mutual knowledge, institutional context, and role-definition give shape to interaction. Simmel’s deceptively simple definition of the stranger as one who is physically proximate but socially distant is instructive here, as is his analysis of the place of mutual indifference in interactions between stranger in cities [Simmel, 1971; see also, Horgan (2012, 2017) and Marotta (2000, 2012)]. For Simmel, strangers are those who share only the most general characteristics, and broadly differentiated only according to membership of general, visually available categories. This observation from Simmel is later more formalized by both Goffman (1983) and Lofland (1973) who note that public space is a distinct realm of interaction where the interaction therein has a peculiar character: it is exclusively based on categorical rather than individual or biographical knowledge.^7^ Consequently, stranger interactions in public space are highly ritualized.

Civil conduct in interaction with strangers is not simply functional. Like all rituals, civil inattention upholds demonstrable membership in a collective, but unlike many other rituals, conditions for inclusion are minimal. In their ideal form, public spaces shared by strangers are broadly egalitarian and freely accessible (Young, 1990). In practice, few public spaces match this ideal. Indeed, decades of research shows the unequal application of civil inattention, with those in structurally vulnerable positions more subject to ritual breaches (Gardner, 1989, 1995; Duneier and Molotch, 1999; Anderson, 2011, 2023). Civil attention is a ritual means for demonstrating a form of inclusion that is intersubjectively rather than legally upheld (Horgan, 2019). In this sense, it is “one of the ways in which we communicate respect for others and generate habits of moral equality” in everyday life (Boyd, 2006, p. 863). Uncivil acts, then, are not simply failures to abide by rules of conduct. They connect to inequality, exclusion, and marginalization. Theoretically, examining accounts of such ritual breaches can build upon and draw together insights from both EMCA and cultural sociology.

## Doing things with accounts of ritual breaches in public space

Breaches are not only naturally occurring phenomena of use to analysts. The stable structure that ritual provides means that any deviation from the ritual form, any failure to uphold its basic structure may become a topic and resource for lay analysis: ritual breach is a locus for lay interpretation. Thus, accounts of ritual breaches provide a switching point between an EMCA focus on the interaction order and a cultural sociological focus on structures of meaning and interpretation. Where EMCA centers members’ attempts at correction *in* interaction, the illustrative data below centers on the organization of interpretive resources members use *about* interaction. When civil inattention—“the slightest of interpersonal rituals…that constantly regulates the social intercourse of persons” (Goffman, 1963, p. 84)—is breached, members have things to say.

To illustrate, we now turn to some illustrative data from interviews with adults in Canada about their ‘most recent encounter with a rude stranger in public space.’ These semi-structured interviews (*n* = 326)^8^ were conducted in-person by the author and student researchers in locations of participants’ choosing. To systematically solicit accounts of uncivil encounters, interviews began by gathering a range of demographic information. Instead of survey-style box-ticking, gender, age, race and ethnicity (and, where participants deemed them relevant, sexuality, religion, and nationality) were recorded in participants’ own words. Probes invited participants to elaborate on their accounts in very fine detail (e.g., spatio-temporal setting, their emotional state, stranger’s appearance and demeanour, phases of the encounter). Having solicited detailed accounts of encounters, interviews then invited participants to reflect on these encounters and on possible justifications for uncivil conduct.

While the specifics of each breach provide accounts’ substance, breaches also provide hooks on which to hang moral interpretations. As Rawls notes, “situated interactional requirements are moral obligations, commitment to which constitutes an implicit social contract with moral implications” (Rawls, 2022, p. 32). Interviews made explicit what is otherwise implicit. Uncivil conduct invites interpretation in a moral register; it induces moral judgment. How, then, do members interpret apparent absence of “mutual commitment to enacted practice” (Rawls, 1996, p. 479) in public space? How do members understand breaches of the ritual of civil inattention, read here as failures to uphold such mutual commitment?

Where EMCA works to specify the patterned sequences of action essential to the achievement of order (Turowetz and Maynard, 2010), my argument concerns how participants interpret and make sense of breaches of the moral order of everyday urban life. Thus, I focus less on the dynamics of the breach itself and the interactional moves involved in repairing and restoring order, instead attending to how participants make sense of these encounters. First, I suggest that participants’ stated understandings of civil conduct position it as a moral imperative oscillating between universal application and individual exception. A universalist moral orientation—kindness—forms the basis for the second theme. Here, participants describe how kindness toward a rude stranger can be invoked to turn encounters into *teachable moments*. Then, picking up on the way that participants’ accounts reach beyond the rude encounter itself, I discuss a cluster of themes centered on treating uncivil encounters as opportunities for three forms of what I call *moral messaging*. While not mutually exclusive—many interviews contain more than one theme—here, they are separated for the purpose of illustration. Taken together, they demonstrate how participants interpret uncivil encounters as morally meaningful.

### Everyday morality between universal application and individual exception

“I do not think it’s OK to be rude ever, um, but of course there’s a thin line between, there’s right and wrong. There’s a morality issue” (Lisa, woman, white, early 20s).^9^

Across accounts, participants orient to conduct in public as being about basic respect, specifically, the rights of all to use public space without being intervened upon unnecessarily. John, a white man in his early twenties: “rudeness is uncalled for, I do not think there’s ever a reason why people should be rude…especially in public spaces, that should be a place where people can be themselves and they should be, you know, not interrogated by other people.” For Erik, another white man in his early twenties: “if you want to be a better person and make an impact on the earth, not like Nobel Prize impact, but just morally be a good person and know that you did well in your life then you have to strive to not be rude to anybody.” Sofia, a young white woman also foregrounded this universal orientation: “if you are true to your morals…you should treat everyone the way you should, you want to be treated and I think that I would like to be treated with respect.”

This universally applied everyday morality appeared across interviews, with many participants making strong claims about the generalized applicability of rules around public conduct. Mo, an Asian man is his early twenties: “I do not think it [incivility] should be justified because everyone has their own agenda. Everyone’s in a hurry. Everyone has things they need to do. What makes my time more important than theirs?.” Similarly, Kaya, a young white woman reflecting on a rude encounter with another young white woman in a crowded public space: “There should not be any reason to treat anyone disrespectfully and there’s obviously better ways to handle situations than being rude to people…it would never be okay to be rude to a stranger. You should always be treating everyone with respect.”

While most participants made universal claims around conduct, many tempered these claims by referencing how personal circumstance may impact conduct. For example, Kate, a middle-aged white woman reflects on a rude encounter with a middle-aged white man: “people should always think through what they say because you do not know who you are talking to and what they are going through.” In a similar vein, but drawing on personal context as providing partial justification, Marie, a young white woman reflecting on a rude encounter with a middle-aged white man, says: “everyone has their own thing going on, their own story, and their own lives…I think if it’s warranting rudeness, then it must be more important than holding a door for me.” Simon, an Asian man in his 20s, notes: “I truly believe everybody deserves respect and kindness…but sometimes it’s just difficult when I’m impatient or I’m going through personal things, and I do not think of anyone else around me, except my own…um…my own agenda and my own self and it becomes that much more difficult to recognize and be sensitive to being nice to other people.”

Universal moral claims, then, can be attenuated by considering an individual’s personal context. ‘Having a bad day’ figured both in excusing incivility, and in participants themselves holding back in being rude to a stranger. Thus, while participants invoked basic universal moral principles in the abstract, they were also willing to understand how the context of actually experienced everyday interaction meant that strict adherence to universal morality may loosen in light of personal circumstance. That said, even if the loosening of strict moral principles was possible, participants were nonetheless clear that the existence of such principles was unquestionable.

### ‘Killing with kindness’: uncivil encounters as teachable moments

In addition to universal moral claims around respect, many participants subscribed to a doctrine of universal kindness. While kindness may be functional in living among others, most participants described it as a moral necessity. Reflecting on a rude encounter with a young Asian man, Zola, a middle-aged African-Canadian woman says: “I do not believe that if one is conscious of a situation there’s ever a reason for [being rude]…I do not think so. I think that being mindful of other people…the better route to take is always kindness.”

When universally applied kindness extended beyond deservingness. The term ‘killing with kindness’ appears regularly in interviews. Here, the uncivil encounter provides an opportunity for members to engage in a *teachable moment*: “I know people who would say if somebody’s rude to you, like I’m going to be rude back to them…I would say that if somebody is rude to you, like, you should just be even nicer to them, so that, like, you are basically killing them with kindness, right?” (Sarah, woman, white, 50s). Asked if rudeness is *ever* justified, Leslie, a woman who had encountered a rude stranger taking up too much space and blocking a door to public transit, says: “even if they are rude to you…fight it with kindness.” More pointedly, Tess, a middle-aged woman who was confronted by a stranger about her dog, offers: “if someone is rude to me…I’ll usually just tell them to have a nice day…‘coz I want them to think of their own behavior.” Here the breach of civil inattention wedges open opportunities to instruct violators about conduct in public by leading with example. This repeats throughout interviews:

“I believe in the philosophy of killing people with kindness so I am just going to be nice to these people and if they are still rude to you it just kind of looks worse on them than the effort will on you…I just think you should be as polite as possible and then if they are still going to be rude to you then it just shows their character not yours.”

“if someone has been rude to you, sure I understand retaliating, but at the end of the day I do not think that rudeness solves anything. I think that you should kill them with kindness.”

As a generalized strategy then, ‘killing with kindness’ allows members to posit themselves as exemplars of moral purity amidst the potential pollution of everyday interaction in public space. The next section shows the depth of the moral vein that runs through accounts.

### Moral messaging: standing up, calling out, and interactional Robin Hoods

Where ‘killing with kindness’ allows participants to position themselves as moral actors, many went further, describing and interpreting uncivil encounters as opportunities to defend collective ideals around the sanctity of persons and of social bonds. Across many interviews, participants drew on uncivil encounters as opportunities for *moral messaging*, especially when asked “is it *ever* justified to be rude to a stranger in public space?”

Here, participants articulated the need to intervene in rude encounters as a form of repair work connected to the protection of sacred ideals, specifically the sacred qualities of persons and of social order. These took three main forms; (i) *standing up* in defense of the sanctity of the self; (ii) *calling out* in defense of the sanctity of others, and (iii) *being an interactional Robin Hood* in defense the sanctity of social order. Where *standing up* and *calling out* concern affronts to personhood, *being an interactional Robin Hood* is concerned with affronts to the moral order of everyday public interaction, and aim at restoring that order.

#### ‘Standing up’: defending the sanctity of self

In discussing encounters involving personal affronts, some participants readily sought to defend themselves. In such cases they justified their own uncivil conduct as standing up for the sanctity of their personhood. Rob, a young white man discussing the conduct of a middle-aged male stranger who approached him in an aggressive manner, says: “if someone were to approach you aggressively or harassed you in anyway, I believe that everyone has the right to defend themselves, but if the stranger is doing nothing wrong then you should not be rude.” Meeting rudeness with rudeness is here justified in defense of self.

This theme of standing up for oneself repeated in many interviews. For example, Fred, a young Asian man recounts a rude encounter with a young white woman, and when asked about justifying uncivil conduct, says: “where someone was demeaning me as a person or my character or was like being threatening in some way, or in my face, you know, just being very aggressive in the way they were speaking to me…I think in that situation, I do not know if I would say being rude was appropriate or being aggressive, but I guess standing up for yourself...like ‘this is not okay, I’m not going to sit here and let you treat me like shit basically’.” In another case, Chad, a white man in his thirties says: “there’s situations where I feel you do not have to be polite, but it’s generally in response to rudeness…if someone does something, there are people out there that you know, they’ll do things in public and you know you should not have to stand for you should not allow people to do sort of whatever they want…if they are impeding you or they are lashing out at you or anything like that I think you have the right to respond how you choose you do not have to be polite. I mean if it’s not your job to take their shit then why would you?”

Meeting like-with-like in defense of one’s personhood was a persistent justification participants offered to rationalize their own rude conduct. Ali, a young south Asian man says: “There might have been times where I was rude to a point that I would stand up for myself if someone is being pushy with me and causes me to be rude to stand up for myself then I might react but I have never been rude to a stranger without a reason. If I’ve ever been rude there must be a reason that caused me in being and acting rude toward a stranger.”

In another account, Case, a young gay man encountered a stranger on a university campus who offered unsolicited commentary on his sexuality: “even though I was hurt by the comments because I am a part of the LGBTQ…community, I still think that um, that I should like leave my emotions aside, and kind of be a role model by not responding to him and you know, maybe by even educating him or people who display other forms of like aggression to other people, whether that be racism, homophobia, or any other um, you know, form of discrimination…think people should maybe educate others, or just not engage with them…those are probably the most appropriate and mature responses.” Here, Case provides multiple anchoring points for justifying rudeness, incorporating both defense of self and others.

#### ‘Calling out’: defending the sanctity of others

“When people are being really rude to me. Or, if say I’m, I’m in a line up and there are people who are being rude to the cashier, I will become very aggressive to protect the underdog” (Jim, middle-aged white man).

Where ‘standing up’ is a form of moral messaging concerned with defending oneself, participants also discussed defending others, or what I refer to here as “calling out.” Patty, a middle-aged white woman says: “unless I’d seen them do something unjust to somebody else but then I think I’d call them out on it, I would not be rude necessarily….if there’s an elderly person standing on the bus and there’s…a bunch of young people or able bodied people sitting down and…able to stand and…nobody offers a seat, then I would probably say something.”

Some participants, though, did express concern that ‘calling out’ may be aimed only at a potentially inauthentic moral performance: “I think with like social media and stuff, I think it’s easy for people to feel more obligated to stand up now, because they want that 5 min of fame of like all the likes on Facebook or like the video views of them standing up to someone being rude.”

Despite concerns around inauthenticity, where uncivil conduct addressed particular categories of persons many participants reported feeling compelled to call rude strangers out. Here, calling out aims at protecting potentially vulnerable persons. Examples offered included defense of women, elderly persons, disabled persons, and members of visible or sexual minorities. Carlos, a middle-aged Latino-Canadian man says: “if someone’s being racist or unjust in any way…sometimes you need to speak up, sometimes you are in a situation where you cannot just walk away and you need, you need to…maybe we can all find ways of speaking up without being rude. You know, no name calling, or you know other rude things.” Kendra, a white woman in her early twenties: “The only time you are allowed to be rude to someone is if, you see them doing something, hateful…like, let us say there’s like a couple fighting and the girl is trying to get away from the guy, and he keeps grabbing her and you are like ‘hey buddy, back off’ and you need to like, get in the middle of them, and get her away and be like…‘what you are doing is wrong’.” Similarly, Parv, a south Asian woman in her early 20s says: “if you saw someone...someone who did not have a handicap parking pass parking in a handicap spot, I think it would be appropriate to say ‘what are you doing? Why are you doing that? That’s not appropriate’...other than that, no.” ‘Calling out’ was readily invoked when uncivil conduct connected to membership of particular categories.

In some accounts ‘calling out’ simply defended individuals in particular situations, in others, it was about defending particular categories of persons whose personhood was being demeaned. Still others more readily connected justification for rudeness as moral messaging centered on defending social order more generally. Marl, a young white woman in reflecting on her own conduct in encounters with rude strangers, offered the following bringing together multiple dimensions of moral messaging:

“I have been border on rude to people who have made homophobic comments to me or have insulted people with mental illness…Rudeness in the face of any sort of homophobia, sexism, racism, any of those types of things, or any injustice, I’m okay with that. I’m okay with being called rude for reacting that way because it’s just not okay…any time when, like if you are watching or see someone being assaulted in any way, that is perfectly fine to be rude to that stranger who’s doing the assaulting. If you are in conversations with people and they are just blatantly being completely disrespectful to another human being or to you based on your gender, your sexuality, your race, anything – yes, it is okay to be rude to that person. That’s completely justified, does not matter if they are working in service, does not matter what they are doing. You have every right to defend yourself in that situation.”

Here two forms of moral messaging—‘standing up’ to defend the sanctity of self, and ‘calling out’ in defense of others—dovetail with one another. This brings us to another form of moral messaging: in defense of social order, or what I call *being an interactional Robin Hood*.

#### Interactional Robin Hoods: defending the sanctity of social order

As we have seen so far, reflecting on uncivil encounters provides opportunities for members to justify their own uncivil conduct in defense of the sanctity of personhood, both self and other. A third form involves participants treating uncivil encounters as interactional resources upon which to hang moral claims around the sanctity of the social bond more generally (Horgan, 2020).

Being an interactional Robin Hood figured most prominently in denser settings, popping up regularly in cases of queue jumping where rude strangers breach the basic rules of distributional justice (Schwartz, 1975). For example, Paul, a young white man spoke about an encounter with another young white man who pushes in front of him at a busy bar. He confronts the queue-jumper, saying: “We’re university students…we are civilized, I get it…it’s a bar, I get it…some people are intoxicated, but we are all civilized enough to know that you stand in line, you wait in line, like you have done it a million times before I’m sure, it’s just common sense…you wait in line.” Lin, a white woman in her 40s confronts a man in a busy parking lot who sped in front of her to take a parking spot: “It unfolded with me getting out of my car in the middle of the parking lot and going and standing at his door before he could get out of the car to tell him that he had taken my parking and how disrespectful and rude it essentially was. And explaining to him that…it’s a little bit concerning for me as an older person to know that this is Canada’s future that’s going to be running the country, people with these norms and morals or lack thereof.” Lin treats the infraction as a signal or symptom of moral decline, to be put right by defending not just herself or another, but the form of distributional justice that pertains in the mundane moral order of a parking lot.

This kind of concern about distributional justice appeared again and again. Anna, a young white woman boards a busy bus and notes a “younger white guy” with headphones on who leaves his bag on the empty seat next to him while many people on the bus are standing: “I approach him and tell him about it and just say that it’s rude, you know, like, our parents like to bring us up in a certain way, you know and follow certain rules, or moral guidelines I guess you could say.” Simon, an Asian man in his 20s recounts the following experience at a busy highway coffee stop with a long lineup, where a white man in his 30s arrives and steps to the top of the line:

“I say ‘I’m telling you right now, that you will not get served here by standing there’. I’m three or four back—but you will not get your coffee before me. And so he went on with some vulgar language, and the ‘person in front of him moved and he orders his coffee. And I said excuse me ma’am, if you serve him a coffee, I’m going to ask everybody’ in this restaurant to leave. This is wrong. He is not next in line…he said something to the tone of ‘I could buy this place if I wanted to’. And he looked at me and pointed his finger, and he said, ‘you are making all this trouble…I could have had my coffee and could have been gone by now’. I say ‘that’s true, and the people in front of me could say the same thing, if you had not been rude and butted in’.”

Across accounts then, participants spoke of the need to intervene to protect the sacred character of collective life. The justifications provided are forms of *moral messaging*. In the case of *standing up*, participants defend the sanctity of their personhood. With *calling out* moral messaging defends the sanctity of another’s personhood. And by *being an interactional* Robin Hood, moral messaging is in service of sacred social order.

## Everyday lifeworlds as moral landscapes

Across the range of themes identified above—universally applied moral principles and their situational attenuation, members’ use of uncivil encounters with strangers in public space as teachable moments and opportunities for moral messaging—we see how ordinary members orient to the everyday lifeworlds of urban public space as deeply moral landscapes. *Landscape* here is explicitly points to co-existence of multiple, potentially contradictory, moral justifications. Like physical landscapes, particular elements are foregrounded or backgrounded (Zerubavel, 2015). Landscapes are available to be interpreted by all who engage them. A moral landscape can contain multiple meanings. Moral landscape highlights not only this polysemy, but also members’ interpretive capacities in foregrounding and backgrounding different kinds of moral justifications, and the interpretive work necessary to make them morally intelligible. While members foreground or background different meanings, a moral landscape is irreducible to those populating it: it has an existence over and above interactions occurring within it. The intelligibility of accounts is oriented to but not wholly organized by the interaction being recounted. Rather, to make accounts intelligible, members seek to provide moral clarity. This necessarily involves foregrounding and backgrounding different elements of the moral landscape of everyday urban lifeworlds.

EMCA attunes us to the eternally ongoing work of intersubjectively negotiating the doing *of* interaction *in* interaction. Members practically accomplish this as a matter of course in everyday life. Members do not treat the ritual structure of stranger interaction as simply functional. Rather, as their accounts of breaches of the everyday stranger interaction ritual of civil inattention suggest, members treat encounters as morally meaningful. While action at the scene of interaction is a kind of ‘doing’ in the ethnomethodological sense of practical accomplishment, *tellings* too are doings. Members’ accounts and justifications mean providing interpretations that are recognizable. By offering accounts in a moral register, participants’ make them more generally intelligible beyond the specifics of any particular encounter. Where EMCA examines accountability and tellability, this has focused on how these are put together *in* interaction. In the case of the second-order accounts *about* interaction gone awry discussed here, I suggest that moral intelligibility draws on a discursive structure aligned with the binary discourse of civil society (Alexander, 2006). Grounded in Durkheim’s basic binary division of the social world, echoing through Goffman, and organized around what people deem to be morally good or bad, this structure provides interpretive resources for understanding infraction *qua* infraction, and for positioning it within a broader moral landscape. As shown in many of the quotes above, the conduct of others is subject to forms of judgment that are not sourced exclusively within the interaction order. Rather, a deeper binary structure provides consistent interpretive resources—sacred/profane, good/bad, care/indifference, kindness/malice—for members to draw upon in making sense of and accounting for encounters in everyday life.

With this basic binary comes a cultural structure readily referencing moral ideals. Such ideals though cannot be reduced to raw empirical fact or generalized to some nebulous abstraction. Rather, they are drawn upon and borne out of reflection on lifeworld experiences: embodied experience provides the tangible reality where moral ideals manifest. As I have suggested, it is, in part, ritual elements of mundane social life that foreground interaction’s moral dimensions, such that breaches may elicit immediate responses in defense of that order at the scene of interaction, and *post hoc* interpretations centering collective life’s moral underpinnings [see also Horgan (2019)]. It is not only in ritualized encounters themselves or in their breaches, but also in reflections upon such encounters that we get some purchase on how everyday life and the broader moral worldviews that swirl around it connect.

The theoretical approach advanced and illustrated here brings fresh eyes to the analytic utility of interactional breaches in EMCA, in social theory, and in sociology more generally. EMCA takes up the Durkheimian tradition of centering social pathologies—interactional breaches—to analyze the production of social order, though it does so to highlight sense-making procedures in everyday life. While few cultural sociologists have taken mundane interactional breaches seriously, they do recognize that breaches can also bring deeper structures of meaning to the surface. As Alexander notes, in “periods of significant social tension and conflict, deeper structures come into play and people draw upon them to experience and transform fundamental meanings of social life. So we can see that underlying sacred structures weave in and out of mundane life” [quoted in Lynch and Sheldon (2013)]. The scene of mundane interaction is one place where social tensions surface, and it may just be that while the kinds of tensions that cultural sociologists take seriously have tended to be more largescale societal ones, this approach brings cultural sociological attention to the more everyday kinds of goings-on of interest in EMCA.

## Conclusion: ties and tensions between EMCA and cultural sociology

At the scene of interaction, ritual is solidarizing, ritual breach, potentially desolidarizing. Interviews consistently illustrate how interaction ritual structure provides opportunity for affiliative or disaffiliative responses, a structure for participants to offer or withdraw solidarity, however minimal or fleeting. Consequently, participants treat breaches as morally meaningful where departures from interactional norms—breaches of the interaction ritual of civil inattention—are taken as *expressive*. For many, such acts reach beyond the immediate act and scene of interaction, while remaining interpretively tethered to it. For members, any given scene of interaction is enmeshed in a wider world. In this sense, ritual is Janus-faced, providing analytic inputs in two directions: facing interactional practices *and* structures of meaning. Similarly, while implicit rules of conduct may be broadly agreed upon, any participant’s interpretation of an encounter cannot be wholly determined by those rules.^10^ The meanings of what happens within the interaction order are not always immediately clear, and this ambiguity can both thicken and lift in breaches of the ritual order of copresent interaction. Interviews suggest that members establish clarity by giving a moral anchoring to their interpretations: interpretations are made intelligible by treating everyday lifeworlds as moral landscapes.

In the foregoing, everyday uncivil encounters provide access to ordinary morality. This is a meeting point of sorts where the spirit of a variety of sociologies springing from Durkheim’s work intersect. Over a century ago, Durkheim noted that when he set out to study society what he uncovered was morality. This fundamental insight is shared by both EMCA and cultural sociological approaches. For the former, morality is made evident at the scene of interaction in the local production of social order as a moral order (Rawls, 2010). For the latter, culturally structured collective representations have some autonomy from any particular interaction order and provide interpretive resources to understand everyday life. While both approaches operate broadly within interpretive traditions distinguishable from sociology’s more critical and straightforwardly positivist traditions, EMCA proceeds through rigorous commitment to empirics, cultural sociologists through a postpositivist approach (Alexander, 1990). EMCA brings close analytic attention to everyday life as a moral order by detailing the practical accomplishment of order in everyday life. These insights are foundational and continue to resonate in EMCA, both conceptually and empirically (Garfinkel, 1967; Rawls, 2010; Jayyusi, 2014). In a similar spirit, centered on interactional practices in everyday life, when we attend to members’ reflections on breaches, we open up new insights. Interview participants readily seek to connect relatively minor infractions in everyday life to bigger, deeper moral issues. While seeding connections, however tenuous, between EMCA and cultural sociology, the present argument also affirms and further nuances EMCA’s foundational tenet: everyday interaction is a moral order that members interpret and defend as such.

Even with shared Durkheimian roots, fundamental tensions remain between an approach that sees the social world as organized at the level of interactional practices and one that posits the existence of broad cultural structures providing interpretive resources for actors to make sense of the world. That these structures are largely implicit does not mean that members are incapable of discerning them. Rather, they are available to be deployed in ways that members find useful in interpreting everyday interaction. What I have sought to demonstrate here is that, for all their oppositions and tensions, commensurabilities between EMCA and, as a major movement in contemporary social theory, the strong program cultural sociology, are worth investigating. While differing on the analytic status of meaning, both approaches place meaning—its’ production, accomplishment, variety, and malleability—at the center of their methods of theorizing.

To close, I offer a programmatic note. First, in terms of connecting EMCA and sociological theory, continuing to probe the various points of resonance and dissonance between EMCA and cultural sociology will bear analytic fruit. Complementarities merit further exploration, especially in attuning cultural sociologists to the interaction order, and further refining EMCA conceptualizations of culture. Furthermore, in cultural sociology Alexander (2006) seeks to understand how solidarity extends to previously excluded groups through a cultural process of ‘civil repair’.^11^ Second, understanding the interactional dynamics and achievement of solidarity between copresent strangers in cities is essential, not only as a theoretical problem of interest to social scientists, but also in the context of continually increasing global urban population.

Interaction alone cannot be fully understood independently of the interpretive resources that ordinary members use to interpret, understand, explain, and respond to the conduct of others, both in the flow of situated interaction, and in their *post hoc* interpretations, explanations, and justifications for their own conduct and that of others. Opening up dialog between EMCA and cultural sociology is a worthy enterprise. As Roy Turner notes “does not the constancy of social change – you cannot step into the same society twice – ensure that there will always need to be a sociological conversation, without closure?” [quoted in Eglin (2018)]. Here, I have barely scratched the surface. It is precisely because members infuse everyday encounters with meaning that they can situate themselves as moral actors within the moral landscapes of everyday lifeworlds. By treating public spaces as sites for morally meaningful encounters between strangers, and by highlighting some interpretive resources that members use to understand such encounters, we can continue to address some of the myopias identified by Goffman at the very outset, and in so doing, renew and thicken connections between EMCA and sociological theory.

## Data availability statement

The datasets presented in this article are not readily available because permission to share was not granted by the REB. Requests to access the datasets should be directed to mhorgan@uoguelph.ca.

## Ethics statement

The studies involving humans were approved by University of Guelph Research Ethics Board. The studies were conducted in accordance with the local legislation and institutional requirements. The participants provided their informed consent to participate in this study.

## Author contributions

The author confirms being the sole contributor of this work and has approved it for publication.

## Footnotes

^1^Moreover, save for a few exceptions [on EMCA, see Smith et al. (2023); on cultural sociology, see Horgan (Forthcoming)], neither approach sustains focus on stranger interactions in public space.

^2^Interestingly, earlier (non-EMCA) work on patient-psychiatrist interaction posits that it is the very ritual structure of the diagnostic encounter that provides opportunities for its’ breach (Laing, 1966).

^3^Moerman’s culturally contexted conversation analysis’ provides another potential route for connecting EMCA and cultural sociology by way of ethnography.

^4^Though, public places where strangers interact are a ‘category rich arena’ [Jayyusi, 2014, p. 265; see also Smith R. J. (2022)].

^5^To date, cultural sociologists have tended to focus on largescale ritual (Schwartz, 1991; Xu, 2009).

^6^In this sense, where Garfinkel’s conceptual touchstone is Schutzian, mine is Durkheimian. For more on the intelligibility/normativity distinction (though with regard to ‘accountability’), see Stevanovic (2023). Thanks to a reviewer and Melisa Stevanovic for helping me to clarify the distinction between my use of breaches and Garfinkel’s.

^7^Smith R. J. (2022, pp. 99–101) discusses the category ‘stranger’ in both Goffman and EMCA.

^8^Data gathering was formally approved by the University of Guelph Research Ethics Board as part of the Researching Incivilities in Everyday Life (RIEL) project and the Sociable Cities Project both funded by Insight Grants from the Social Sciences and Humanities Council (Canada). Transcriptions generated a corpus in excess of 500,000 words. See Horgan (2020) for further details and analysis of the dataset.

^9^Names have been changed and any potentially identifying removed to protect the anonymity of interview participants. Demographic information is as self-reported by interview participants.

^10^As Cicourel (1980, p. 18) notes “the status of normative rules during social interaction still remains unclear in social interaction.”

^11^While this term refers to the macro-level dynamics through which historical wrongs may be amended, the term itself borrows from the EMCA idea of ‘repair after next turn’ (Schegloff, 1992). Alexander (2006) indicates that his concept of ‘civil repair’ grew out of engagement with Schegloff while both were faculty at UCLA (Alexander, personal communication).

## References

[ref1] Abrutyn S. (2022). Disintegration in the age of COVID-19: biological contamination, social danger, and the search for solidarity. Am. Behav. Sci.:00027642221132176. doi: 10.1177/00027642221132176

[ref2] Alasuutari P. (2023). Conversation analysis, institutions, and rituals. Front. Sociol. 8:1146448. doi: 10.3389/fsoc.2023.1146448, PMID: 37881646 PMC10595141

[ref3] Alexander J. C. (1990). Beyond the Epistemological Dilemma: General Theory in a Postpositivist Mode. Sociol. Forum. 5: 531–44. Available at: http://www.jstor.org/stable/684684.

[ref6] Alexander J. C. (2006). The civil sphere. New York; Oxford: Oxford University Press.

[ref7] Alexander J. C. Smith P. (2001). “The strong program in cultural sociology” in The handbook of sociological theory (New York: Kluwer)

[ref8] Alexander J. C. Smith P. (2005). The Cambridge companion to Durkheim. Cambridge, UK; New York: Cambridge University Press.

[ref9] Anderson E. (2011). The cosmopolitan canopy: Race and civility in everyday life. New York: Norton.

[ref10] Anderson E. (2023). Black in white space: the enduring impact of color in everyday life. Chicago: University of Chicago Press.

[ref11] Arminen I. A. T. Heino A. S. M. (2023). Civil inattention—on the sources of relational segregation. Front. Sociol. 8:1212090. doi: 10.3389/fsoc.2023.1212090, PMID: 37731909 PMC10508291

[ref12] Bergmann J. R. (1993). Discreet indiscretions: the social Organization of Gossip, New Brunswick Transaction: Publishers.

[ref13] Bergmann J. R. (1998). Introduction: morality in discourse. Res. Lang. Social Interact. 31, 279–294. doi: 10.1080/08351813.1998.9683594

[ref9008] Boyd R. (2006). ‘The Value of Civility?’ Urban Studies. 43, 863–78.

[ref14] Blair-Loy M. (2009). Competing devotions: career and family among women executives. Cambridge: Harvard University Press.

[ref15] Burke K. (1969). A grammar of motives. Berkeley: University of California Press.

[ref16] Cicourel A. V. (1980). Language and social interaction: philosophical and empirical issues. Sociol. Inq. 50, 1–30. doi: 10.1111/j.1475-682X.1980.tb00015.x

[ref18] Corte U. Parker J. N. Fine G. A. (2019). The microsociology of creativity and creative work. Soc. Psychol. Q. 82, 333–339. doi: 10.1177/0190272519881629

[ref19] DeLand M. F. (2021). Men and their moments: character-driven ethnography and interaction analysis in a park basketball rule dispute. Soc. Psychol. Q. 84, 155–176. doi: 10.1177/01902725211004894

[ref20] Dingemanse M. Roberts S. Baranova J. Blythe J. Drew P. Floyd S.. (2015). Universal principles in the repair of communication problems. PLoS One 10:e0136100. doi: 10.1371/journal.pone.0136100, PMID: 26375483 PMC4573759

[ref21] Duneier M. Molotch H. (1999). Talking City trouble: interactional vandalism, social inequality, and the “urban interaction problem”. Am. J. Sociol. 104, 1263–1295. doi: 10.1086/210175

[ref22] Durkheim Émile. (1964). The rules of sociological method. New York: Free Press.

[ref23] Durkheim Émile. (1995). The elementary forms of religious life. Trans. Karen Fields. New York: Free Press.

[ref24] Eglin P. (2018). Roy Turner (1928 to 2017): a preliminary appreciation. Can. Rev. Sociol. 55, 325–332. doi: 10.1111/cars.12204

[ref25] Eliasoph N. Lichterman P. (2003). Culture in interaction. Am. J. Sociol. 108, 735–794. doi: 10.1086/367920

[ref27] Fine G. A. (2010). The sociology of the local: action and its publics. Sociol Theory 28, 355–376. doi: 10.1111/j.1467-9558.2010.01380.x

[ref28] Fine G. A. (2012). Group culture and the interaction order: local sociology on the Meso-level. Annu. Rev. Sociol. 38, 159–179. doi: 10.1146/annurev-soc-071811-145518

[ref29] Fine G. A. Fields C. D. (2008). Culture and microsociology: the anthill and the veldt. Annals Am. Acad. Pol. Soc. Sci. 619, 130–148. doi: 10.1177/0002716208320138

[ref9001] Francis D. Hester S. (2017). Stephen Hester on the Problem of Culturalism. J. Pragmat. 118, 56–63. doi: 10.1016/j.pragma.2017.05.005

[ref30] Gardner C. B. (1989). Analyzing gender in public places: rethinking Goffman’s vision of everyday life. Am. Sociol. 20, 42–56. doi: 10.1007/BF02697786

[ref31] Gardner C. B. (1995). Passing by: Gender and public harassment. Berkeley: University of California Press.

[ref32] Garfinkel H. (1967). Studies in ethnomethodology. Englewood Cliffs: Prentice Hall.

[ref33] Garfinkel H. (2002). “Ethnomethodology’s program: working out Durkheim’s aphorism” in Rawls. ed. Warfield A. (Lanham, Md: Rowman & Littlefield)

[ref34] Goffman E. (1956). The nature of deference and demeanor. Am. Anthropol. 58, 473–502. doi: 10.1525/aa.1956.58.3.02a00070

[ref35] Goffman E. (1963). Behavior in public places: notes on the social Organization of Gatherings. New York: Free Press.

[ref36] Goffman E. (1967). Interaction ritual: essays on face-to-face behavior. New York: Anchor Books.

[ref37] Goffman E. (1971). Relations in public: Microstudies of the public order. New York: Harper Row.

[ref38] Goffman E. (1983). The interaction order. Am. Sociol. Rev. 48, 1–17. doi: 10.2307/2095141

[ref42] Horgan M. (2012). Strangers and Strangership. J. Intercult. Stud. 33, 607–622. doi: 10.1080/07256868.2012.735110

[ref43] Horgan M. (2017). “Mundane Mutualities: solidarity and strangership in everyday urban life” in Place, diversity and solidarity. eds. Oosterlynck S. Schuermans N. Loopmans M. (New York: Routledge), 19–32.

[ref44] Horgan M. (2019). Everyday incivility and the urban interaction order: theorizing moral affordances in ritualized interaction. J. Lang. Aggress. Confl. 7, 32–55. doi: 10.1075/jlac.00018.hor

[ref45] Horgan M. (2020). Urban interaction ritual: Strangership, civil inattention and everyday incivilities in public space. Pragmatics 30, 116–141. doi: 10.1075/prag.19022.hor

[ref46] Horgan M. (2021). Sacred civility? An alternative conceptual architecture informed by cultural sociology. J. Politeness Res. 17, 9–33. doi: 10.1515/pr-2020-0031

[ref47] Horgan M. (Forthcoming). “The Civil Sphere in Everyday Life: Public Space and the Boundaries of Civil Inclusion” in The Civil Sphere in Canada. eds. Alexander J. C. Mervyn H. (Vancouver: UBC Press)

[ref9003] Inglis D. Thorpe C. (2023). Beyond the ‘Inimitable’ Goffman: From ‘Social Theory’ to Social Theorizing in a Goffmanesque Manner. Frontiers in Sociology 8:1171087. doi: 10.3389/fsoc.2023.1171087 38024785 PMC10630919

[ref48] Ickes W. J. (2009). Strangers in a strange lab: how personality shapes our initial encounters with others. Oxford: Oxford University Press.

[ref49] Jayyusi L. (2014). Categorization and the moral order. London: Taylor and Francis.

[ref51] Kendon A. (1990). Conducting interaction: patterns of behavior in focused encounters. Cambridge: Cambridge University Press.

[ref52] Kuhn T. (1962). The structure of scientific revolutions. Chicago: University of Chicago Press.

[ref53] Laing R. D. (1966). Ritualization and abnormal behaviour. Philos. Trans. R. Soc. Lond. B Biol. Sci. 251, 331–335.

[ref54] Lareau A. (2011). Unequal childhoods class, race, and family life. Berkeley: University of California Press.

[ref55] Levinson S. (2006). “On the human ‘interaction engine’” in Roots of human sociality: culture, cognition and interaction. eds. Enfield N. J. Levinson S. C. (Princeton, New York: Berg), 39–69.

[ref56] Lichterman P. (2020). How civic action works: Fighting for housing in Los Angeles. Princeton University Press.

[ref57] Lichterman P. Eliasoph N. (2014). Civic action. Am. J. Sociol. 120, 798–863. doi: 10.1086/679189

[ref58] Lofland L. (1973). A world of strangers: order and action in urban public space. New York: Basic Books.

[ref59] Lofland L. (1998). The public realm: exploring the city’s quintessential social territory. New York: Aldine De Gruyter.

[ref60] Lynch G. Sheldon R. (2013). The sociology of the sacred: a conversation with Jeffrey Alexander. Cult. Relig. 14, 253–267.

[ref61] Marotta V. (2000). The stranger and social theory. Thesis Eleven 62, 121–134. doi: 10.1177/0725513600062000008

[ref62] Marotta V. (2012). Georg Simmel, the stranger and the sociology of knowledge. J. Intercult. Stud. 33, 675–689. doi: 10.1080/07256868.2012.739136

[ref63] Maynard D. W. (2012). “Everyone and no one to turn to: intellectual roots and contexts for conversation analysis” in Handbook of conversation analysis. eds. Sidnell J. Stivers T. (London: Wiley), 9–31.

[ref64] Maynard D. W. Clayman S. E. (1991). The diversity of ethnomethodology. Annu. Rev. Sociol. 17, 385–418. doi: 10.1146/annurev.so.17.080191.002125

[ref65] Moerman M. (1988). Talking culture: ethnography and conversation analysis. Philadelphia: University of Pennsylvania Press.

[ref66] Mondada L. (2009). Emergent focused interactions in public places: a systematic analysis of the multimodal achievement of a common interactional space. J. Pragmat. 41, 1977–1997. doi: 10.1016/j.pragma.2008.09.019

[ref67] Mondada L. Bänninger J. Bouaouina S. A. Camus L. Gauthier G. Hänggi P.. (2020). Human sociality in the times of the Covid-19 pandemic: a systematic examination of change in greetings. J. Socioling. 24, 441–468. doi: 10.1111/josl.12433 38607822 PMC7536989

[ref68] Mondada L. Peräkylä A. (2024). New perspectives on Goffman in language and interaction: body, participation and the self. London: Routledge.

[ref70] Rawls A. W. (1996). Durkheim’s epistemology: the neglected argument. Am. J. Sociol. 102, 430–482. doi: 10.1086/230952

[ref71] Rawls A. W. (2009). An essay on two conceptions of social order: constitutive orders of action, objects and identities vs. aggregated orders of individual action. J. Class. Sociol. 9, 500–520. doi: 10.1177/1468795X09344376

[ref72] Rawls A. W. (2010). “Social order as moral order” in Handbook of the sociology of morality. eds. Hitlin S. Vaisey S. (New York: Springer), 95–121.

[ref73] Rawls A. W. (2012). Durkheim’s theory of modernity: self-regulating practices as constitutive orders of social and moral facts. J. Class. Sociol. 12, 479–512. doi: 10.1177/1468795X12454476

[ref74] Rawls A. W. (2022). Situating Goffman’s ‘interaction orders’ in Durkheim’s social fact lineage. Grounding an alternate sociology of modernity in heightened awareness of interaction. Etnogr. Ric. Qual. 1, 27–62. doi: 10.3240/103744

[ref75] Rawls A. W. Turowetz J. (2021). ‘Discovering culture’ in interaction: solving problems in cultural sociology by recovering the interactional side of parsons’ conception of culture. Am. J. Cult. Sociol. 9, 293–320. doi: 10.1057/s41290-019-00079-6

[ref76] Revers M. (2023). Performative polarization: the interactional and cultural drivers of political antagonism. Cult. Sociol. doi: 10.1177/17499755231188808

[ref9006] Sacks H. (1995). Lectures on Conversation. Oxford: Blackwell.

[ref77] Schegloff E. A. (1992). Repair after next turn: the last structurally provided defense of intersubjectivity in conversation. Am. J. Sociol. 97, 1295–1345. doi: 10.1086/229903

[ref78] Schegloff E. A. (2006). “Interaction: the infrastructure for social institutions, the natural ecological niche for language, and the arena in which culture is enacted” in Roots of human sociality: culture, cognition and interaction. eds. Enfield N. J. Levinson S. C. (New York: Berg), 70–96.

[ref79] Schwalbe M. Godwin S. Holden D. Schrock D. Thompson S. Wolkomir M. (2000). Generic processes in the reproduction of inequality: an interactionist analysis. Soc. Forces 79, 419–452. doi: 10.2307/2675505

[ref80] Schwartz B. (1975). Queuing and waiting: studies in the social organization of access and delay. Chicago: University of Chicago Press.

[ref81] Schwartz B. (1991). Mourning and the making of a sacred symbol: Durkheim and the Lincoln assassination. Soc. Forces 70, 343–364. doi: 10.2307/2580243

[ref82] Smith G. (2022). “Ritual” in The Routledge international handbook of Goffman studies. eds. Jacobsen M. H. Smith G. (London: Routledge), 38–50.

[ref9004] Simmel G. (1971). “The Stranger.” In On Individuality and Social Forms. ed. Levine D.. (Chicago: University of Chicago Press), 143–49.

[ref9005] Smith P. (2020). Durkheim and after: The Durkheimian Tradition, 1893-2020. Cambridge: Polity.

[ref83] Smith R. J. (2022). “Interaction in public places” in The Routledge international handbook of Goffman studies. eds. Jacobsen M. H. Smith G. (London: Routledge), 97–107.

[ref84] Smith R. J. Ablitt J. Williams J. Hall T. (2023). The coining of convivial public space: homelessness, outreach work, and interaction order. Urban Plan. 8, 42–51. doi: 10.17645/up.v8i4.6457

[ref85] Smith P. Phillips T. L. King R. D. (2010). Incivility: the rude stranger in everyday life. Cambridge: Cambridge University Press.

[ref87] Stevanovic M. (2023). Accountability and interactional inequality: the Management of Problems of interaction as a matter of cultural ideals and ideologies. Front. Sociol. 8:1204086. doi: 10.3389/fsoc.2023.1204086, PMID: 37404337 PMC10315850

[ref88] Stevanovic M. Olakivi A. Nevalainen H. Henttonen P. Ravaja N. (2023). Telling a supervisor about experiences of gendered dismissal: problems of documentation, tellability, and failed authority. Gender Work Organ. 31:22. doi: 10.1111/gwao.13088

[ref89] Stivers T. (2015). Coding social interaction: a heretical approach in conversation analysis? Res. Lang. Soc. Interact. 48, 1–19. doi: 10.1080/08351813.2015.993837

[ref90] Stivers T. Enfield N. J. Brown P. Englert C. Hayashi M. Heinemann T.. (2009). Universals and cultural variation in turn-taking in conversation. Proc. Natl. Acad. Sci. 106, 10587–10592. doi: 10.1073/pnas.0903616106, PMID: 19553212 PMC2705608

[ref91] Tavory I. (2022). “Occam’s razor and the challenges of generalization in ethnomethodology” in The ethnomethodology program. eds. Maynard D. W. Heritage J. (Oxford: Oxford University Press), 420–441.

[ref92] Tavory I. Fine G. A. (2020). Disruption and the theory of the interaction order. Theory Soc. 49, 365–385. doi: 10.1007/s11186-020-09384-3

[ref93] Tavory I. Swidler A. (2009). Condom semiotics: meaning and condom use in rural Malawi. Am. Sociol. Rev. 74, 171–189. doi: 10.1177/000312240907400201

[ref94] Terkourafi M. Kádár D. Z. (2017). “Convention and ritual (Im)politeness” in The Palgrave handbook of linguistic (Im)politeness. eds. Culpeper J. Haugh M. Kádár D. (London: Palgrave), 171–195.

[ref95] Turner V. W. (1995). The ritual process: structure and anti-structure. New York: De Gruyter.

[ref96] Turowetz J. J. Maynard D. W. (2010). “Morality in the social interactional and discursive world of everyday life” in Handbook of the sociology of morality. eds. Hitlin S. Vaisey S. (New York: Springer), 503–526.

[ref97] Valentino L. Vaisey S. (2022). Culture and durable inequality. Annu. Rev. Sociol. 48, 109–129. doi: 10.1146/annurev-soc-030320-102739

[ref98] Willis P. E. (1981). Learning to labor: how working class kids get working class jobs. New York: Columbia University Press.

[ref99] Wilson T. P. Zimmerman D. H. (1979). Ethnomethodology, sociology and theory. Humboldt J. Soc. Relat. 7, 52–88.

[ref100] Wuthnow R. (1989). Meaning and moral order: Explorations in cultural analysis. Berkeley: University of California Press.

[ref101] Xu B. (2009). Durkheim in Sichuan: the earthquake, National Solidarity, and the politics of small things. Soc. Psychol. Q. 72, 5–8. doi: 10.1177/019027250907200102

[ref102] Young I. M. (1990). Justice and the politics of difference. Princeton: Princeton University Press.

[ref103] Zerubavel E. (2015). Hidden in plain sight: the social structure of irrelevance. Oxford: Oxford University Press.

